# An investigation of the role acceptor side chains play in the processibility and efficiency of organic solar cells fabricated from small molecular donors featuring 3,4-ethylenedioxythiophene cores†

**DOI:** 10.1039/c8ra07034b

**Published:** 2018-11-23

**Authors:** N. A. Mica, S. A. J. Almahmoud, L. Krishnan Jagadamma, G. Cooke, I. D. W. Samuel

**Affiliations:** Organic Semiconductor Centre, SUPA, School of Physics and Astronomy St Andrews Fife KY16 9SS UK; Glasgow Centre for Physical Organic Chemistry (GCPOC), WestCHEM, School of Chemistry, University of Glasgow Glasgow G12 8QQ UK

## Abstract

Organic photovoltaic devices fabricated from small molecular donors continue to receive significant interest due to their desirable properties such as convenient synthesis, purification and batch-to-batch reproducibility. In this study, we have synthesized two small molecules based on an alternating A–D–A structure, utilizing a central EDOT donor moiety and either 2-ethylhexyl cyanoacetate (SAM-72) or *N*-(2-ethylhexyl)cyanoacetamide (SAM-80) units as acceptor termini. The small molecules were incorporated into bulk heterojunction solar cells with PC_71_BM. Our investigations have shown that the side chains utilized for SAM-80 only allow for solution processing using volatile solvents, such as chloroform, which limits the reproducibility of device fabrication. However, SAM-72 displays better solubility and devices fabricated using a SAM-72:PC_71_BM active layer reached average power conversion efficiencies of 1.9%, with fill factors reaching 60%. Post-processing methods such as thermal and solvent vapor annealing were found to significantly increase the stability of devices, but were not able to improve overall device performance.

## Abstract 2

The chemical nature of the acceptor side chain plays an important role in the processability and photovoltaic performance of EDOT-based small molecule donors.

## Graphical abstract

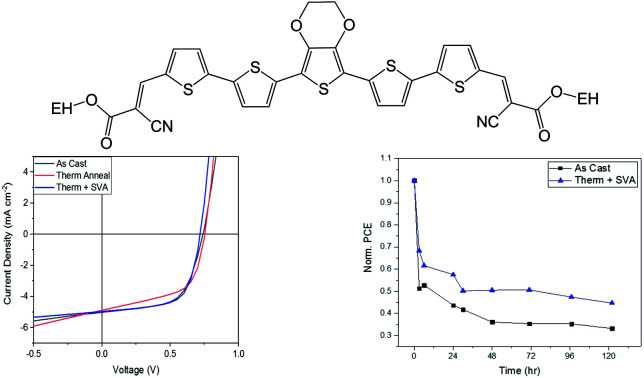

## Introduction

The application of organic materials as the active component of solar cells has received significant attention in view of their increasing power conversion efficiencies (PCEs) and has provided organic photovoltaic (OPV) devices as promising alternatives to silicon based solar cells.^1,2^ Conducting polymers have been widely utilized in bulk heterojunction (BHJ) devices as the donor (D) material, and have given rise to solar cells with PCEs exceeding 11%.^3^ However, the poor batch to batch reproducibility and limited opportunity to develop structure–property relationships of polymeric systems has led to the utilization of small molecules as the active components of BHJs as they offer a range of advantages including well-defined chemical structures, reproducible and scalable synthesis and convenient purification.^4,5^ Moreover, small molecule-based OPVs have reached PCEs comparable with those of polymeric OPVs (∼10%).^6^ Small molecule donors based on oligothiophenes,^7,8^ diketopyrrolopyrroles,^9,10^ triphenylamine,^11,12^ and boron-dipyrromethenes,^13–15^ have received considerable attention, and have been incorporated in alternating donor (D) and acceptor (A) architectures connected by π-conjugated units, *e.g.* D–A–D^16–18^ and A–D–A.^19–24^ This architecture results in a narrow optical band gap and good light harvesting ability,^21,25^ and some A–D–A systems have shown good device performance with fill factors (FF) approaching 70%.^6,26–37^

**Scheme 1:**
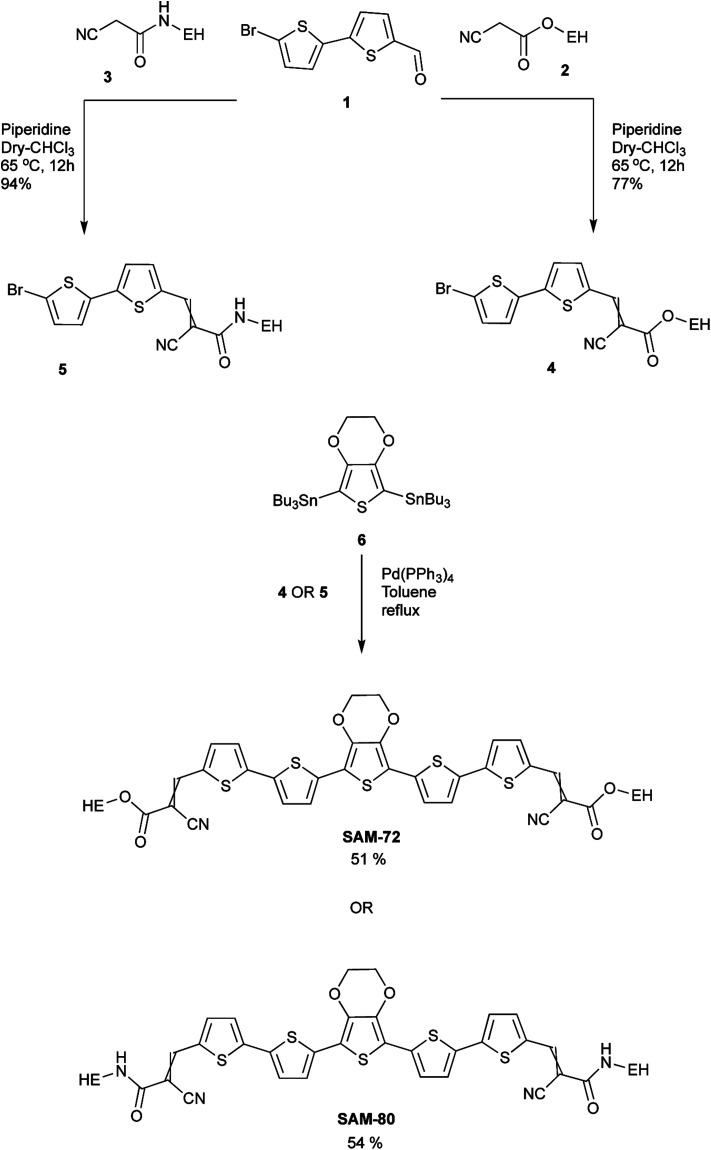
Synthetic route of SAM-72 and SAM-80 (EH = 2-ethylhexyl).

3,4-Ethylenedioxythiophene (EDOT) is a well-studied building block for the development of π-conjugated materials and has been incorporated into small molecule and polymeric systems with OPV applications.^2,38^ The two electron donating oxygen moieties at β positions of the thiophene ring increase the electron donating ability compared to the parent heterocycle whilst simultaneously giving rise to non-covalent interactions (sulfur–oxygen) thereby increasing the rigidification of the π-conjugated system.^39^ Although EDOT has been widely used as a π-linker in D–A type dyes in dye sensitized solar cells (DSSC),^40–45^ to the best of our knowledge, EDOT-based small molecules for BHJ devices have largely utilized a D–A type architecture,^2,44,46^ and relatively little has been focused on A–D–A architectures.^47,48^ In this article, we report the synthesis of symmetrical A–D–A based systems featuring electron rich EDOT cores and their application as solution processed BHJ solar cells with the acceptor [6,6]-phenyl C_71_-butyric acid methyl ester (PC_71_BM). The molecules were designed to feature EDOT as the central donor moiety with adjacent thiophene π-conjugated units to increase the electron donating ability of the donor component. We have utilized 2-ethylhexylcyanoacetate (SAM-72) and *N*-(2-ethylhexyl)cyanoacetamide (SAM-80) as the terminal acceptor groups, which have been previously shown to be promising terminal acceptor moieties.^8,49–52^ The chemical structure of the molecules are shown in Scheme 1. The cyanoacetamide unit was included in this study due to the propensity of the amide unit to participate in hydrogen bonding, which may modulate the packing structure *via* intermolecular interactions and may promote self-assembly of the small molecules within their thin films, resulting in the enhancement of fill factor (FF) and power conversion efficiency. Devices were then fully optimized by various post-processing methods such as thermal and solvent vapor annealing.^53,54^ We also present a qualitative discussion about the morphology of the BHJs and the stability of these solar cells in ambient conditions.

## Results and discussion

### Synthesis

The synthesis of the target molecules SAM-72 and SAM-80 is shown in Scheme 1. Compound 2 was synthesized using a reported procedure of a similar compound.^55^ Compounds 3 (ref. 56) and 6 (ref. 57) were prepared using known procedures. Knoevenagel condensation of compound 1 with the two acceptors 2 and 3 gave compounds 4 and 5 which were coupled to the reagent 6 to give the final molecules SAM-72 and SAM-80 in 51% and 54% yield, respectively.

### Theoretical calculations

**Fig. 1:**
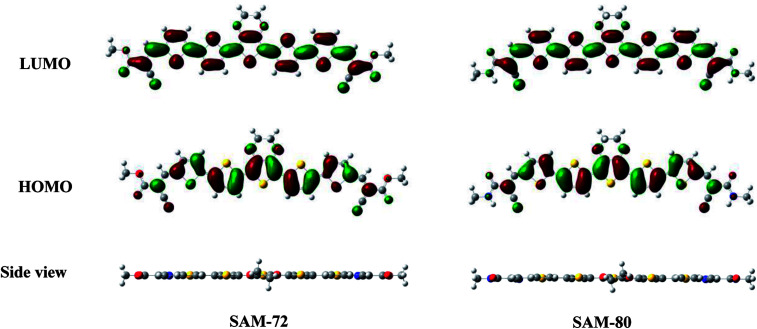
HOMO/LUMO maps of SAM-72 and SAM-80 predicted by DFT calculations.

DFT calculations were conducted to study the electronic structure of SAM-72 and SAM-80. Ground state optimized geometry in Fig. 1 gave rise to planar structure for both molecules, with delocalized HOMO/LUMOs located over the molecular backbone. This planarity is an important feature as it promotes stacking interactions *via* π–π interactions, resulting in a material that can form good film quality with high molecular order.^45^ HOMO, LUMO and *E*_g_ energy levels estimated by DFT calculations are summarized in Table 1.

**Table 1:** Theoretical, optical and electrochemical data of SAM-72 and SAM-80

Molecule	Theoretical calculations			Optical properties				Electrochemical properties		
	HOMO (eV)	LUMO (eV)	*E*_g_ (eV)	*λ*_max_ (nm)	*λ*_onset_ (nm)	*E*_opt_ (eV)	*ε* (L mol^−1^ cm^−1^)	IP (eV)	EA (eV)	*E*_fund_ (eV)
SAM-72	−5.46	−3.41	2.05	548	647	1.82	88 700	−5.08	−3.20	1.88
SAM-80	−5.36	−3.27	2.09	536	629	1.97	58 600	−5.10	−3.14	1.96

### Optical properties

**Fig. 2:**
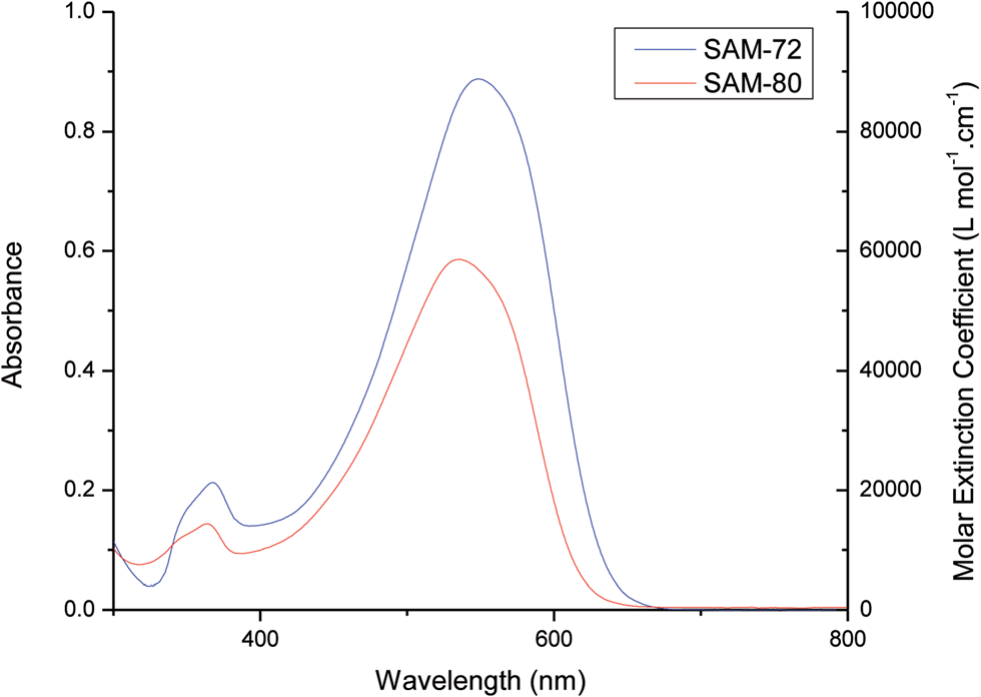
UV-Vis absorption spectra of SAM-72 and SAM-80 in DCM (1 × 10^−5^ M).

Fig. 2 shows the UV-Vis absorption spectra of SAM-72 and SAM-80 in dichloromethane (DCM) solution, and their absorption properties are summarized in Table 1. Both molecules have a very similar absorption profile with absorption onset (*λ*_onset_) at 647 nm and 629 nm for SAM-72 and SAM-80, respectively. SAM-72 shows a maximum absorption (*λ*_max_) at 548 nm, and a slight bathochromic shift (∼12 nm) compared to SAM-80. This suggests that the cyanoacetate terminal group has a slightly stronger accepting property than cyanoacetamide group. The optical band gap (*E*_g_) of SAM-72 (1.82 eV) is also found to be 0.15 eV smaller than SAM-80 (1.97 eV). SAM-72 also shows a strong absorption in the visible region with a molar extinction coefficient (*ε*) of 88 700 L mol^−1^ cm^−1^.

### Electrochemical properties

**Fig. 3:**
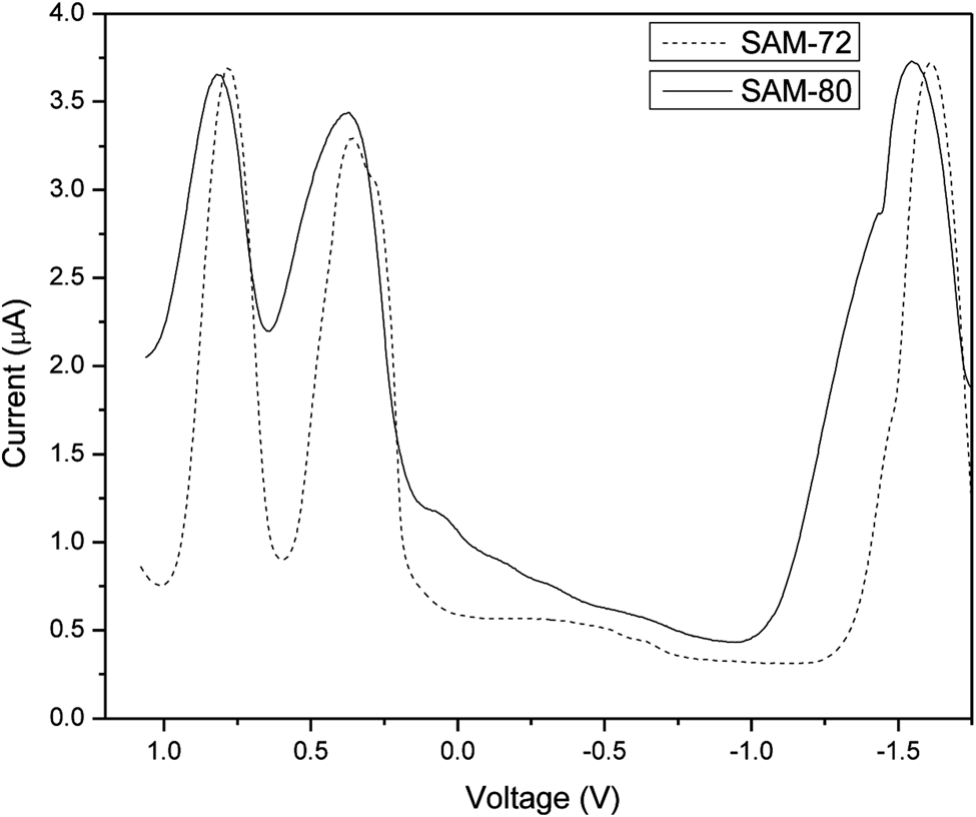
Square-wave voltammograms of SAM-72 and SAM-80 in DCM (1 × 10^−3^ M) (V *vs.* Fc/Fc^+^).

The electrochemical properties of SAM-72 and SAM-80 were determined by square wave voltammetry and the data are summarized in Table 1. Both molecules show two oxidation waves at reasonably low potentials (Fig. 3). Ionization potentials (IP), electron affinity (EA), and fundamental band gap (*E*_fund_) were estimated from the oxidation and reduction potentials, respectively, and showed that the *E*_fund_ of SAM-80 is ∼0.08 eV larger than that of SAM-72. SAM-80 also shows lower IP (−5.10 eV) compared to SAM-72. This suggests that the change in terminal acceptors from cyanoacetamide to cyanoacetate increases the HOMO/IP level which can change the open circuit voltage (*V*_oc_) in OPV devices, as *V*_oc_ is proportional to the difference between the HOMO level of the donor and the LUMO level of the acceptor (*i.e.*, PC_71_BM).^58,59^

### Device application and optimisation

After the synthesis of these donor materials, the next step was to test their photovoltaic performance in solar cells by analyzing their current–voltage (*J*–*V*) curves and external quantum efficiency (EQE) spectra. Initially SAM-80 and SAM-72 were tested by making solutions in chloroform with the fullerene derivative PC_71_BM. These devices showed promise with PCEs reaching an average of 1.78% for SAM-80 (1 : 3 wt%) and 1.16% for SAM-72 (1 : 5 wt%). The corresponding *J*–*V* characteristics and OPV performance parameters are shown in ESI Fig. S1 and Table S1.† However, due to the volatile nature of chloroform, active layers formed from this solvent were not reproducible and higher boiling-point solvents needed to be used. For this we tried using either chlorobenzene or dichlorobenzene. SAM-80 was not able to reliably dissolved in these solvents, whereas SAM-72 presented excellent solubility in chlorobenzene. In order to investigate whether intermolecular hydrogen bonding (N–H⋯O=C) interactions of the acrylamide moieties were responsible for the lack of solubility of SAM-80, we have performed solid-state FT-IR and solution ^1^H NMR spectroscopy on this material. The IR study provided a N–H stretch (major) at 3371 cm^−1^ (Fig. S2†) which is consistent with hydrogen bonding interactions occurring in the solid-state.^60^ Dilution NMR experiments performed in CDCl_3_, on the other hand, did not show any evidence for N–H participation in hydrogen bonding and indicated that π–π stacking interactions are more significant (Fig. S3†).

**Fig. 4:**
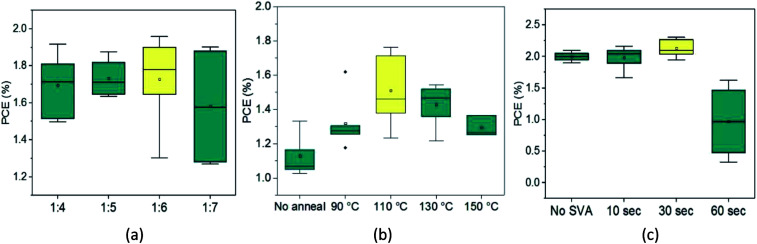
Box plots for each optimization step for SAM-72:PC_71_BM devices:(a) donor to acceptor ratio, (b) thermal annealing temperature, and (c) thermal annealing + SVA time. In each graph the average of the distribution is represented by the square in the box plot, the median is a horizontal line in the box, the ends of the box represent the data within the 25% to 75% percentile, the whiskers extending from the box are the range within 1.5 standard deviations, and the outliers are the black dots outside the boxes.

With reliable solubility, SAM-72 and PC_71_BM were combined in a 10 mg mL^−1^ solution using chlorobenzene. Investigation of the photovoltaic properties started with the optimization of donor to acceptor ratio in the BHJ blend solution. Due to the device performance of this blend favoring high amounts of acceptor (1 : 5 wt%) when using chloroform as the casting solvent, donor to acceptor weight ratios ranging from 1 : 4 to 1 : 7 were blended and used for BHJ formation using chlorobenzene. As seen in Fig. 4a, and in ESI Table S2 and Fig. S4,† the 1 : 6 ratio was observed to result in the highest average PCE, and was chosen to be the optimum weight ratio. When increasing the acceptor amount in the blend from 1 : 4 to 1 : 6 an increase in device FF and overall PCE was observed. This trend stops when using the 1 : 7 weight ratio blend, where we observe a drop in FF and decrease in current from 3.69 mA cm^−2^ to 3.55 mA cm^−2^.

BHJ processing methods such as thermal annealing,^53,61–63^ solvent vapor annealing (SVA),^54,64,65^ and the use of additives^66–68^ have been studied in detail by various groups and shown to affect device performance. Both thermal annealing and SVA will allow the molecules to break out of their present thermodynamically unfavorable state, and rearrange themselves into a more energetically stable orientation. It has also been shown that using an additive can drastically alter donor or acceptor domain size by causing the materials to swell and reorganize.^66–68^ This adjustment to the domains in the photoactive layer can boost device performance by enhancing the exciton diffusion length or creating domains of optimum size to combat geminate charge recombination.^69,70^ In an attempt to increase the OPV efficiency, a combination of the listed processing methods were applied to the active layer.

To investigate the effect of thermal annealing on the photovoltaic properties of the SAM-72:PC_71_BM blend (1 : 6 wt%), the BHJ films were annealed at different temperatures ranging from 90–150 °C. The PCE of the BHJ OPV devices as a function of thermal annealing is shown in Fig. 4b. The corresponding *J*–*V* characteristics and EQE spectra are shown in ESI Fig. S5 and Table S3.† Annealing the BHJ up to 110 °C led to an increase in FF and short circuit current (*J*_sc_), but higher annealing temperatures resulted in a decrease in these characteristics. From these initial studies of thermal annealing temperature optimization, annealing at 110 °C for 10 minutes is the optimum condition for SAM-72:PC_71_BM films.

After identifying the optimum thermal annealing temperature, BHJ films were exposed to carbon disulfide (CS_2_) vapors to undergo SVA. This solvent was chosen based on its success in SVA applications with other small molecule and fullerene BHJs.^71–73^ Specific details of the SVA procedure are included in the Experimental section. As seen from Fig. 4c, SVA was conducted for four different lengths of time with the highest PCE resulting from a 30 second exposure. The corresponding *J*–*V* characteristics and EQE are shown in ESI Fig. S6 and Table S4.† Exposing this BHJ to the CS_2_ environment for up to 30 seconds can improve device performance, mainly by increasing the FF. But, if left to SVA for longer amounts of time then the current density and FF decrease and lead to a lower PCE.

When performed in sequence, thermal annealing and SVA can produce solar cells that have the maximum *J*_sc_ of 4.34 mA cm^−2^ and an average PCE of 1.90% for this device structure, with the best device performing at 2.31% efficiency. This low amount of current produced in these devices likely comes from the film consisting mostly of fullerene – an acceptor which does not help device current due to its weak absorption peak in the lower-wavelength region of the visible spectrum. Another aspect hindering performance is the thickness of the active layer. When fully optimized the film is 50–60 nm thick, limiting the amount of light that the film can absorb.^61^ To increase this thickness the organic solution concentration was increased from 10 to 20 mg mL^−1^. However, full devices had efficiencies of average 1.1% and the fill factor dropped significantly to <50%. An effort was made to further improve device performance by using the additive 1,8-diiodooctane (DIO) in varied volume percentages in the solution used to make the active layer. However, any amount of DIO caused the formation of large domains not capable of working in a full device (ESI Fig. S7†).

**Fig. 5:**
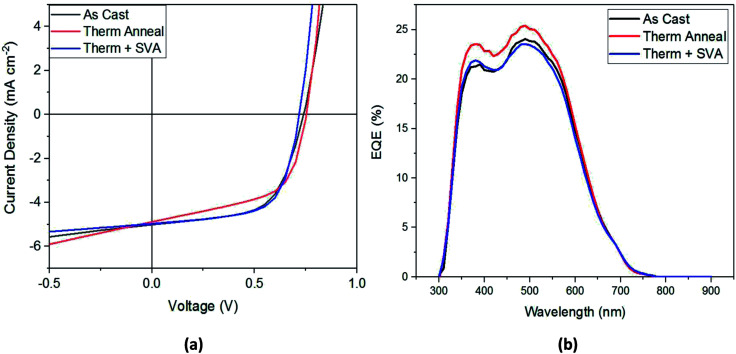
(a) Current–voltage plots and (b) EQE spectra for best performing SAM-72:PC_71_BM devices.

Shown in Table 2, the application of these processing methods did not statistically improve device performance, although individual optimization experiments did show some improvement from the treatments. The best-performing device *J*–*V* curve for each processing method is shown in Fig. 5a. From this figure we can confirm the results of Table 2: thermal annealing results in the lowest FF and highest *V*_oc_, the combination of thermal and SVA leads to the lowest *V*_oc_ but high FF and *J*_sc_, and that any of these post-processing treatments results in devices that perform similarly to as-cast devices. An EQE spectrum for the different types of post-production treatments is shown in Fig. 5b. This figure shows that the thermally annealed film has the best EQE, with its maximum reaching 25% at 500 nm.

**Table 2:** Device performance for SAM-72:PC_71_BM blends for various types of processing. All errors in this table are the standard deviation of the solar cell characteristic distributions

	Best PCE (%)	PCE (%)	FF (%)	*J*_sc_ (mA cm^−2^)	*V*_oc_ (V)
As cast	2.25	1.85 ± 0.17	61.08 ± 2.24	4.13 ± 0.35	0.74 ± 0.01
Thermal	2.10	1.83 ± 0.09	54.17 ± 1.79	4.52 ± 0.26	0.75 ± 0.01
Therm. + SVA	2.31	1.90 ± 0.16	60.45 ± 2.96	4.34 ± 0.26	0.72 ± 0.02

When comparing the literature on other A–D–A molecule solar cells, the device performance for SAM-72:PC_71_BM is competitive with that of other EDOT-based molecules.^44,47,48,74,75^ For example, the material reported by Antwi *et al.* (DECA-2TE), with equivalent side chains and a similar core, showed *J*_sc_ values reaching an average of 2.99 mA cm^−2^ and FF of 39%.^47^ Likewise, another group of EDOT-based materials reported in 2013 gave solar cell device performances with all FF values < 50%.^44^ Similarly, in the EDOT-based molecules reported by Montcada *et al.* in 2013 (ref. 74) and 2015,^48^ only two out of the six donor molecules studied resulted in solar cells with FF > 60%. In this context we believe that SAM-72, with FF > 60% and *J*_sc_ > 4.00 mA cm^−2^, stands as a competitive molecule for OPV applications of EDOT-based molecules.

### Morphology studies with AFM

**Fig. 6:**
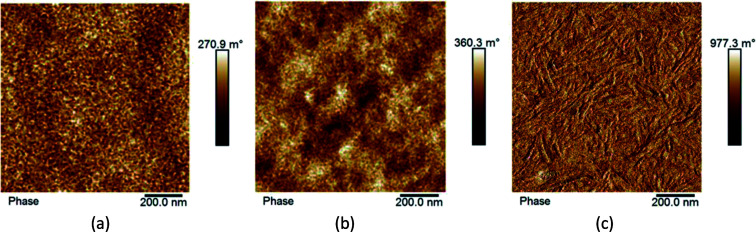
AFM phase contrast images of SAM-72:PC_71_BM films under different processing conditions: (a) as cast film with optimized donor to acceptor ratio; (b) thermal annealed film; (c) film that was both thermally and solvent vapor annealed.

In organic solar cells the level of performance is directly influenced by the morphology of the bulk heterojunction. Here, in Fig. 6, we present phase contrast AFM images of SAM-72:PC_71_BM films when subjected to different post-processing methods. As analyzed from the topographic AFM images presented in ESI Fig. S8,† initial as-cast films show well-mixed donor and acceptor domains with a surface roughness of 3.34 nm. This roughness is reduced to 0.78 nm and the domains begin to aggregate together when annealed at 110 °C for 10 minutes, and then form fiber-like structures when introduced to CS_2_ vapor for 30 seconds with a surface roughness of 0.67 nm.

As discussed above, processing methods such as thermal or solvent vapor annealing will cause the molecules in organic films to order themselves in a lower-energy state, changing the donor and acceptor domain structures.^66–68^ This is observed here (Fig. 6b), as the fullerene domains begin to grow upon thermal annealing. Further optimization by SVA led to the formation of fiber-like structures. Previously these fibers have signified an increase in film crystallinity with P3HT:PCBM blends,^64,67^ and it is assumed here that this processing treatment also induced crystallinity.

### Solar cell operational stability at 1 sun intensity

**Fig. 7:**
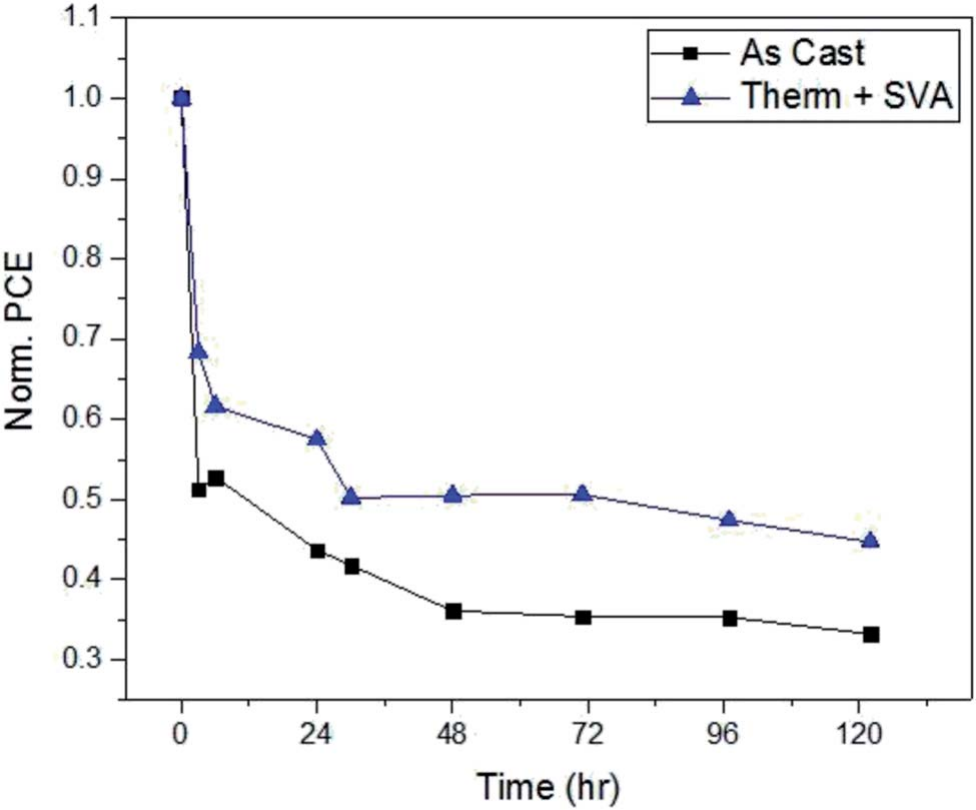
Ambient photo-stability of SAM-72:PC_71_BM devices.

As a test of the stability of the devices, as-cast and fully post-processed solar cells were left in front of 1 sun illumination in ambient conditions for 120 hours and their performance measured at various intervals. Fig. 7 presents the PCE of these devices as a function of time. After an initial burn-in, as cast devices stabilize to an efficiency of approximately 35% of their initial value, while fully post-processed devices retain 50% of their PCE. Although device performance stays roughly the same when additional processing methods are applied to the films, the operational stability is improved as evident from Fig. 7. All devices were encapsulated before being taken out of a nitrogen environment into ambient conditions, therefore it is unlikely that water and/or oxygen was the main source of this degradation. Therefore, a morphology change in the active layer under illumination is what most likely leads to the degradation of these devices. Under illumination, the materials within the BHJ will begin to migrate and form pure domains of donor or acceptor.^76^ If this results in large domains then excitons generated within the domains will recombine before reaching an interface, leading to a decreased PCE. However, different morphologies of the initial BHJ, such as enhanced crystallinity,^76–79^ could have molecular packing that favors a more photo-stable film that will not undergo such domain growth.

To investigate this argument, we compare the atomic force microscope images of as cast and fully optimized films of SAM-72:PC_71_BM in Fig. 6a and c. The crystalline domains that form after SVA will make the molecules more confined and chemical reactions less likely to occur – allowing for the avoidance of photo-chemical reactions which hinder the solar cell performance.^76–79^ Another way in which the film protects itself from these harmful reactions is by becoming denser following thermal annealing.^80^ In the devices presented here, spectroscopic ellipsometry measurements show that undergoing thermal and solvent vapor annealing reduces the active layer thickness by 12.5%. The as-cast films have an initial thickness of 58 nm and decrease to 50 nm when fully processed. With confirmed density and suspected crystallinity increase, the confinement of the small molecule SAM-72 in a BHJ with PC_71_BM is more photo-stable after it is thermally and solvent vapor annealed.

## Conclusions

Here we report the synthesis of small molecule donors SAM-72 and SAM-80 featuring centrally located EDOT units and their incorporation into BHJ solar cells. We have shown that the differing side chains play a significant role in their processability, with SAM-80 being the least processible.^81^ On the other hand, SAM-72 which lacks the amide functionality had much better solubility which allowed BHJ cells to be fabricated with PC_71_BM. Optimization of the donor to acceptor weight ratio provided PCEs of 1.85% and FFs of >60%. Further active layer optimizations such as thermal and solvent vapor annealing do have a substantial impact on BHJ morphology, but do not change device performance overall and the primary impact of these post-processing methods is on the device photo-stability. After a quick burn-in period of device performance under illumination, the stability was improved by 15–20% when the film was thermally and solvent vapor annealed. Although only modest PCEs were obtained, the convenient synthesis of these materials earmark them as a promising class of materials for fabricating organic solar cells.

## Experimental

### General

All reagents are purchased and used as received unless stated otherwise. Dry solvents were obtained from solvent purification system (activated alumina columns) (Pure Solv 400-5-MD), apart from chloroform which was purchased from Sigma Aldrich. Mass spectrometry and elemental analysis were obtained from the mass spectrometry service at the University of Glasgow. Melting points (mp) were recorded on a SMP10 Stuart Scientific melting point machine and are uncorrected. ^1^H NMR and ^13^C NMR spectroscopy were recorded on Bruker AVIII (400 MHz) spectrometer, operating at 400 MHz and 100 MHz, respectively. Chemical shifts are given in ppm and are relative to TMS, all *J* values are in Hz. UV-Vis measurements were carried out using a Perkin Elmer Lambda 25 spectrometer. Square wave voltammetry was recorded at room temperature under nitrogen on CH-instruments 440A potentiostat using a three electrodes cell with a platinum (Pt) working electrode, a Pt wire counter electrode and an Ag wire pseudo reference electrode. Samples were analyzed at 1.0 mM concentrations with a scan rate of 0.1 V s^−1^ using TBAPF_6_ (0.1 M in corresponding solvent) as the supporting electrolyte. The reduction potentials are referenced to ferrocene (internal or external reference) with the Fc/Fc^+^ redox couple adjusted to 0.0 V.

### Computational

Density functional theory (DFT) calculations were performed using a Gaussian 09 software. Molecular geometries were initially optimized semi-empirically (AM1) and then re-optimized by DFT using the B3LYP method with the 6-311Gdp basis set. The absence of transition states were confirmed by the absence of imaginary frequencies in vibrational frequency calculations. The 2-ethylhexyl- side chains were replaced by methyl units to aid the convergence of the geometry optimizations.

### Synthesis

#### Compound 4

5′-Bromo-2,2′-bithiophene-5-carboxaldehyde 1 (0.500 g, 1.83 mmol), compound 2 (0.540 g, 2.75 mmol) and MgSO_4_ (6.00 mg) were introduced to dry CHCl_3_ (20 mL). After the addition of 3 drops of piperidine, the reaction mixture was stirred overnight at 65 °C. The reaction mixture was cooled to room temperature and washed with water (3 × 50 mL). The collected organic extract was dried over MgSO_4_, filtered and concentrated under reduced pressure. The crude product was purified with silica column chromatography using a mixture of petroleum ether and DCM (2 : 1) as eluent. Compound 4 was isolated as a yellow solid (0.78 g, 94%). Mp 84–85 °C. *δ*_H_ (400 MHz, CDCl_3_) 8.24 (1H, s), 7.65 (1H, d, *J* 4.0), 7.18 (1H, d, *J* 4.0), 7.14 (1H, d, *J* 3.9), 7.05 (1H, d, *J* 3.9), 4.22 (2H, dd, *J* 5.8, 1.9), 1.70 (1H, m), 1.39 (8H, m), 0.92 (6H, m). *δ*_C_ (100 MHz, CDCl_3_) 162.9, 146.0, 145.9, 138.8, 137.2, 134.6, 131.3, 126.5, 124.5, 115.7, 114.5, 98.5, 68.9, 38.7, 30.3, 28.9, 23.7, 22.9, 14.0, 11.0. HRMS *m*/*z* (EI+) [M^+^] 451.0273 (requires 451.0275 for C_20_H_22_BrNO_2_S_2_).

#### Compound 5

5′-Bromo-2,2′-bithiophene-5-carboxaldehyde 1 (1.20 g, 4.39 mmol), compound 3 (1.03 g, 5.27 mmol) and MgSO_4_ (6.00 mg) were introduced to dry CHCl_3_ (30 mL). After the addition of 3 drops of piperidine, the reaction mixture was stirred overnight at 65 °C. The reaction mixture was cooled to room temperature and washed with water (3 × 50 mL). The collected organic extract was dried over MgSO_4_, filtered and concentrated under reduced pressure. The crude product was purified with silica column chromatography using a mixture of petroleum ether and DCM (3 : 1) as eluent and compound 5 was isolated as a yellow solid (1.53 g, 77%). Mp 115–116 °C. *δ*_H_ (400 MHz, CDCl_3_) 8.33 (1H, s), 7.58 (1H, d, *J* 4.0), 7.16 (1H, d, *J* 4.0), 7.12 (1H, d, *J* 3.9), 7.04 (1H, d, *J* 3.9), 6.20 (1H, t, *J* 5.8), 3.36 (2H, td, *J* 6.1, 2.1), 1.54 (1H, m), 1.41–1.25 (8H, m), 0.92–0.89 (6H, m). *δ*_C_ (100 MHz, CDCl_3_) 160.5, 144.7, 144.2, 138.1, 137.4, 135.1, 131.41, 126.3, 124.6, 117.4, 114.3, 100.0, 43.64, 39.56, 31.12, 29.01, 24.39, 23.11, 14.20, 11.01. HRMS *m*/*z* (EI+) [M^+^] 450.0462 (requires 450.0435 for C_20_H_23_BrN_2_OS_2_).

#### Compound SAM-72

Compound 6 (0.110 g, 0.153 mmol) and compound 4 (0.152 g, 0.337 mmol) were dissolved in dry toluene (3 mL) and flushed with argon for 15 minutes. After that, Pd(PPh_3_)_4_ (0.009, 0.008 mmol) was added and the mixture was heated under reflux overnight. The resulting dark mixture was cooled to room temperature, then poured into DCM (20 mL) and washed with water (3 × 20 mL). The collected organic extract was dried over MgSO_4_, filtered and concentrated under reduced pressure. The dark crude product was then purified by column chromatography using petroleum ether and THF as eluent (2 : 1), and the collected product was recrystallized from MeOH. SAM-72 was isolated as a dark solid (0.07 g, 51%). Mp 195–198 °C. *δ*_H_ (400 MHz, CDCl_3_) 8.23 (2H, s), 7.64 (2H, d, *J* 4.0), 7.33 (2H, d, *J* 4.0), 7.23 (2H, d, *J* 4.0), 7.17 (2H, d, *J* 4.0), 4.48 (4H, s), 4.21 (4H, dd, *J* 5.8, 1.5), 1.71 (2H, m), 1.50–1.30 (16H, m), 0.96–0.93 (12H, m). *δ*_C_ (100 MHz, CDCl_3_) 163.2, 147.5, 145.9, 139.3, 138.6, 136.4, 134.1, 134.0, 126.8, 124.0, 123.9, 116.0, 110.3, 97.3, 68.8, 65.1, 38.8, 30.3, 28.9, 23.7, 22.9, 14.0, 11.0. HRMS *m*/*z* (FAB+) [M^+^] 884.2128 (requires 884.2116 for C_46_H_48_N_2_O_6_S_5_).

#### Compound SAM-80

Compound 6 (0.500 g, 0.694 mmol) and compound 5 (0.786 g, 1.74 mmol) were dissolved in dry toluene (24 mL) and flushed with argon for 15 minutes. After that, Pd(PPh_3_)_4_ (0.040 g, 0.0347 mmol) was added and the mixture was heated under reflux overnight. The resulting dark mixture was cooled to room temperature, then poured into DCM (20 mL) and washed with water (3 × 20 mL). The collected organic extract was dried over MgSO_4_, filtered and concentrated under reduced pressure. The dark crude product was then purified by column chromatography using petroleum ether and THF as eluent (2 : 1), and the collected product was recrystallized from MeOH. SAM-80 was isolated as a dark solid (0.33 g, 54%). Mp 223–226 °C. *δ*_H_ (400 MHz, CDCl_3_) 8.33 (2H, s), 7.59 (2H, d, *J* 4.0), 7.32 (2H, d, *J* 4.0), 7.22 (2H, d, *J* 4.0), 7.18 (2H, d, *J* 4.0), 6.20 (2H, t, *J* 5.8), 4.48 (4H, s), 3.39–3.34 (4H, m), 1.55 (2H, m), 1.43–1.26 (16H, m), 0.91 (12H, m). *δ*_C_ (100 MHz, CDCl_3_) 160.7, 146.1, 144.0, 138.5, 136.1, 134.4, 134.3, 126.4, 124.0, 123.9, 117.5, 110.2, 98.8, 65.1, 43.4, 39.4, 30.9, 28.8, 24.2, 22.9, 14.0, 10.8. HRMS *m*/*z* (FAB+) [M^+^] 882.2454 (requires 882.2436 for C_46_H_50_N_4_O_4_S_5_).

### Device fabrication

All active layer materials were mixed in chlorobenzene for at least 6 hours at 60 °C. Fully dissolved solutions were then filtered using a 0.45 μm PTFE filter (GE Healthcare). Standard structure devices were all made with the following materials: ITO/PEDOT:PSS/active layer/Ca (10 nm)/Al (80–100 nm). Substrates with an ITO strip were first cleaned thoroughly by detergent (Hellmanex III), acetone, isopropanol, and lastly plasma ashing (GaLa Instrumente GmbH) with O_2_ gas. Cleaned substrates were then coated by PEDOT:PSS (Heraeus Clevios P VP AI 4083) filtered through 0.45 μm PVDF syringe tip (Millex) and spun on at 4000 rpm for 60 seconds, followed by 20 minutes of annealing at 130 °C. Devices were then transferred into a nitrogen-filled glovebox where the active layer was spun on at 900 rpm for 60 seconds. At this point the Ca/Al contact was evaporated on under a vacuum of 10^−6^ mbar at rates of 0.05 nm s^−1^ for both materials. This was followed by encapsulation using an optical epoxy (Norland Optical Epoxy) and subsequent drying under UV light for 60 seconds.

### Device optimisation

For thermal annealing optimization, the hotplate was allowed to reach the desired temperature and allowed to equilibrate for at least 5 minutes before annealing film. Solvent vapor annealing was done by lining a glass Petri dish (7 cm diameter) with CS_2_, placing the film in the center of the dish, and then covering with another dish for a controlled amount of time. The solvent additive DIO was added to the active layer solution 1 hour prior to spin coating to allow for thorough mixing.

### *J*–*V* measurement

Finished devices were taken out of the glovebox into ambient conditions and put under an AM 1.5 solar simulator. An external voltage was applied across the solar cell and resulting current was measured by a Keithley 2400 source meter.

### Stability measurement

Device stability measurements were conducted by leaving samples under ambient conditions and AM 1.5 illumination for the duration and measuring their *J*–*V* curves each time.

## Conflicts of interest

There are no conflicts to declare.

## Supplementary Material

RA-008-C8RA07034B-s001
