# Supplementary material for: An investigation of the role acceptor side chains play in the processibility and efficiency of organic solar cells fabricated from small molecular donors featuring 3,4-ethylenedioxythiophene cores
Source: RSC Adv. 2018 Nov 23;8(69):39231–40. doi: 10.1039/c8ra07034b (PMC9090895; doi:10.1039/c8ra07034b)
Supplement: RA-008-C8RA07034B-s001 [file RA-008-C8RA07034B-s001.pdf]

Electronic Supplementary Material (ESI) for RSC Advances.
This journal is © The Royal Society of Chemistry 2018

Electronic Supplementary Information

An investigation of the role acceptor side chains play in the processibility and efficiency of organic solar cells fabricated from small molecular donors featuring 3,4-ethylenedioxythiophene cores.

N. A. Mica,[a†] S. A. J. Almahmoud,[b†] L. K. Jagadamma,[a] G. Cooke[b] and I. D. W. Samuel*[a]*

[a]Organic Semiconductor Centre, School of Physics and Astronomy, SUPA, St Andrews, Fife, KY16 9SS, UK.

[b]Glasgow Centre for Physical Organic Chemistry (GCPOC), WestCHEM, School of Chemistry, University of Glasgow, Glasgow, G12 8QQ, UK.

†*These authors contributed equally.*

Supporting Figures

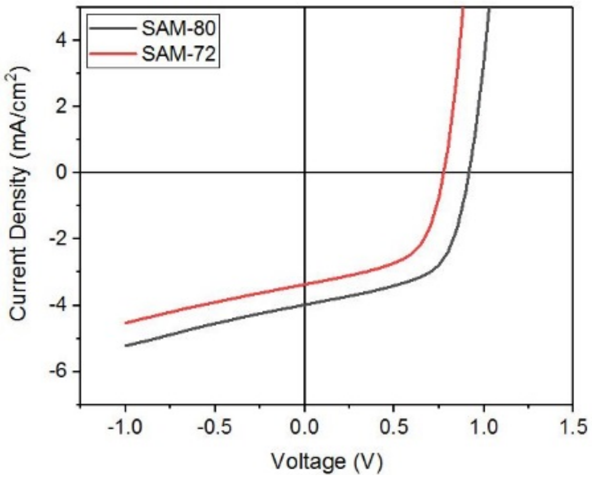

Figure S 1. Current-voltage curves of **SAM-80:PC$_{71}$BM** (1:3 wt%) and **SAM-72:PC$_{71}$BM** (1:5 wt%) devices cast from chloroform.

	Best PCE (%)	PCE (%)	FF (%)	J_{sc} (mA/cm^2)	V_{oc} (V)
SAM-80:PC$_{71}$BM (1:3 wt% in chloroform)	**2.10**	**1.78 ± 0.28**	**58.10 ± 0.59**	**3.36 ± 0.54**	**0.91 ± 0.01**
SAM-72:PC$_{71}$BM (1:5 wt% in chloroform)	**1.46**	**1.16 ± 0.21**	**55.90 ± 0.82**	**2.69 ± 0.49**	**0.77 ± 0.00**

Table S 1. Solar cell performance for the optimum **SAM-80:PC$_{71}$BM** and **SAM-72:PC$_{71}$BM** devices when cast in chloroform.

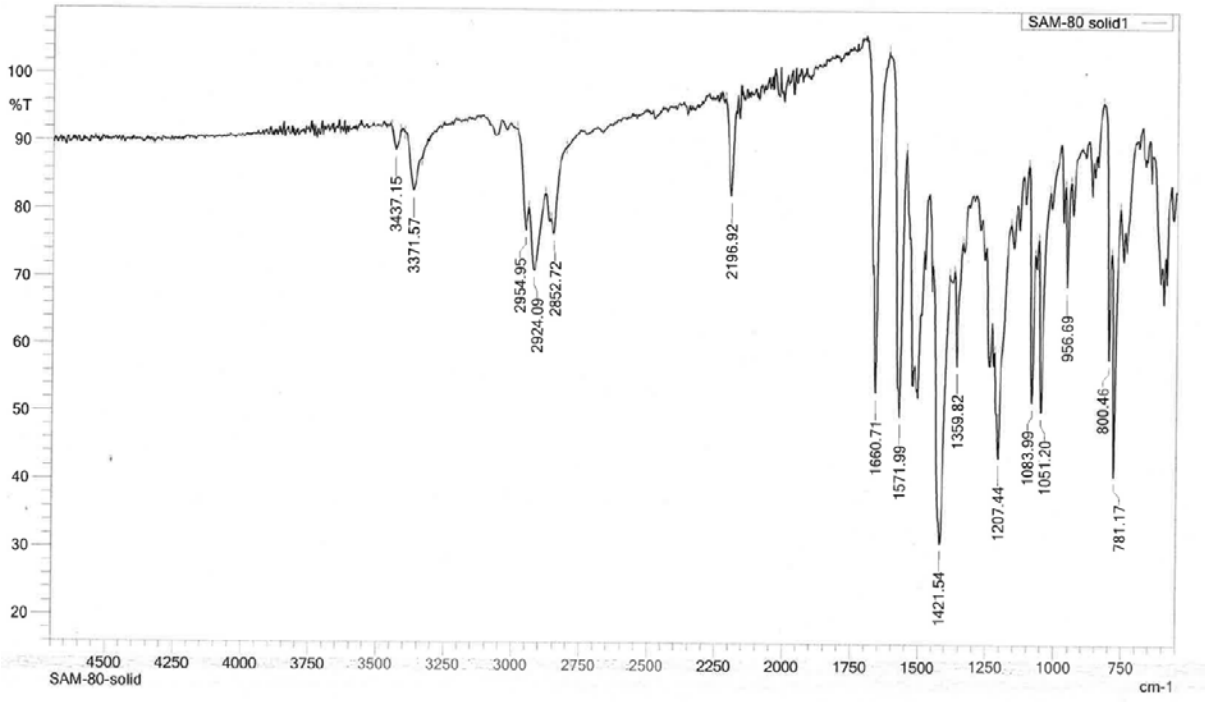

Figure S 2. Solid-state FT-IR spectrum of **SAM-80**. Recorded on IRAffinity-1S Fourier Transform Infrared Spectrophotometer.

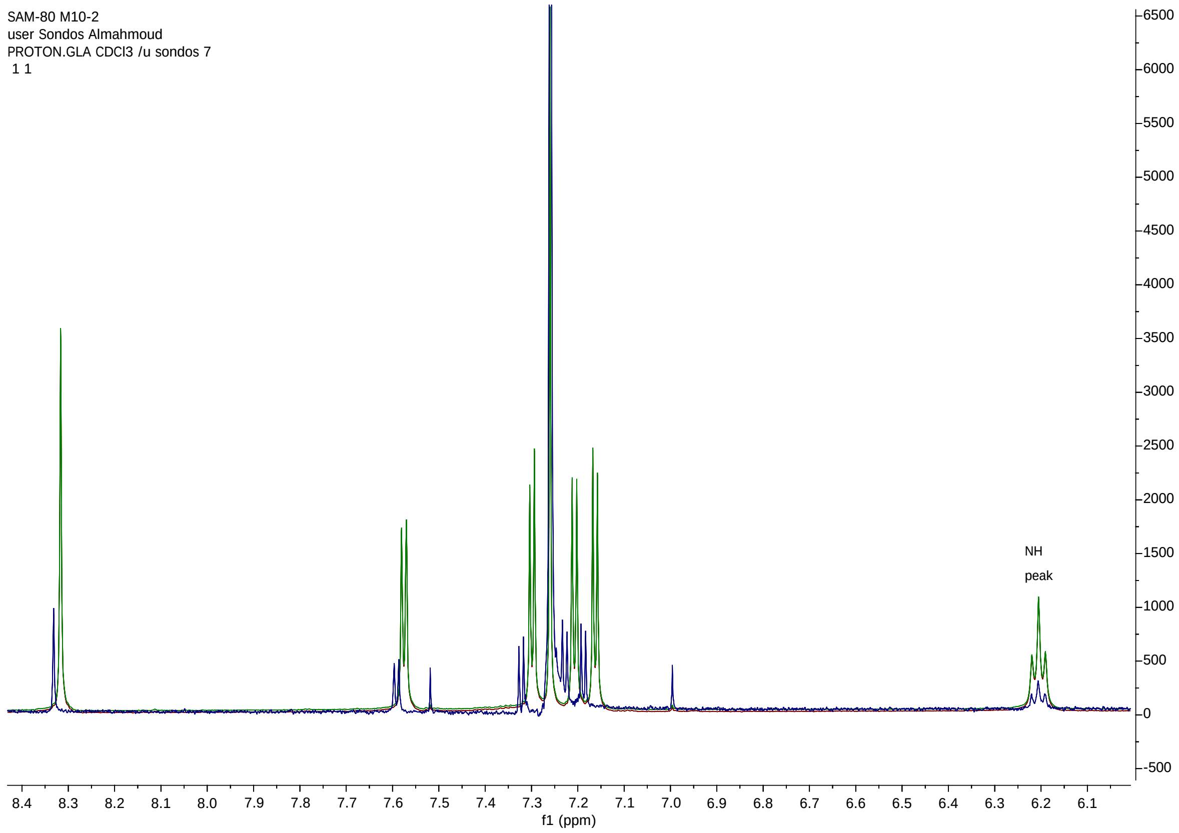

Figure S 3. ^{1}H NMR of **SAM-80** in $CDCl_3$ using two concentrations 10^{-2} M (green) and 10^{-4} M (blue).

D/A weight ratio	Best PCE (%)	PCE (%)	FF (%)	J_{sc} (mA/cm^2)	V_{oc} (V)
1:4	**1.92**	**1.69 ± 0.16**	**59.70 ± 0.66**	**3.83 ± 0.34**	**0.74 ± 0.01**
1:5	**1.88**	**1.73 ± 0.11**	**63.50 ± 1.17**	**3.74 ± 0.17**	**0.73 ± 0.01**
1:6	**1.96**	**1.73 ± 0.24**	**65.20 ± 0.70**	**3.69 ± 0.45**	**0.72 ± 0.02**
1:7	**1.90**	**1.58 ± 0.35**	**61.70 ± 3.70**	**3.55 ± 0.53**	**0.72 ± 0.01**

Table S 2. Solar cell performance for donor to acceptor weight ratio optimization for **SAM-72:PC$_{71}$BM** devices.

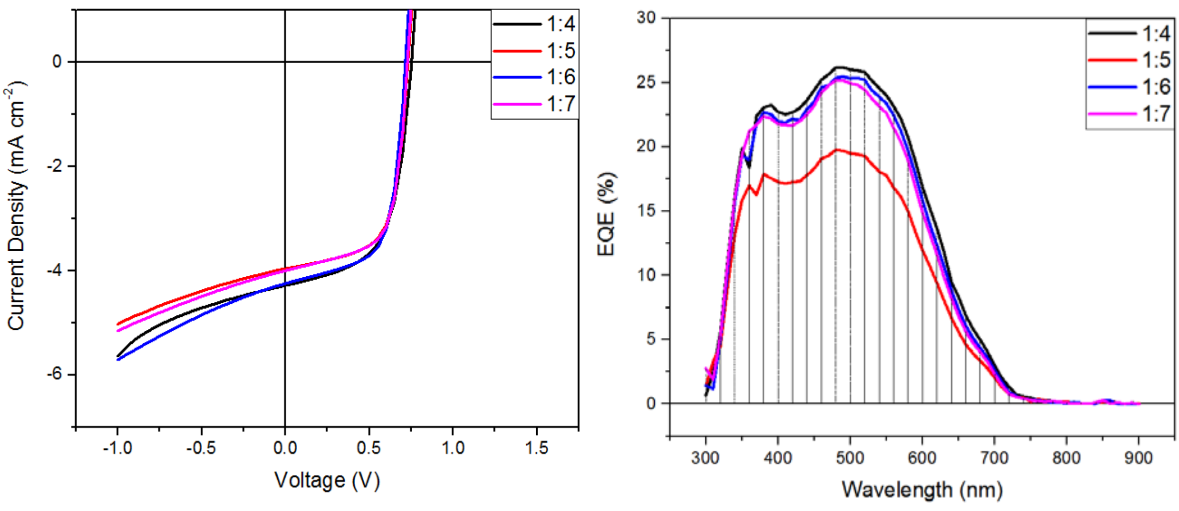

(a) (b)

Figure S 4. (a) Current-voltage plots and (b) EQE spectra for donor to acceptor weight ratio optimization for **SAM-72:PC$_{71}$BM** devices.

Thermal annealing temp (°C)	Best PCE (%)	PCE (%)	FF (%)	J_{sc} (mA/cm^2)	V_{oc} (V)
No anneal	**1.33**	**1.13 ± 0.12**	**47.35 ± 2.66**	**3.40 ± 0.20**	**0.70 ± 0.01**
90	**1.62**	**1.32 ± 0.15**	**48.96 ± 6.29**	**3.72 ± 0.20**	**0.73 ± 0.01**
110	**1.76**	**1.51 ± 0.22**	**52.88 ± 5.27**	**3.86 ± 0.35**	**0.74 ± 0.01**
130	**1.54**	**1.43 ± 0.13**	**53.18 ± 1.36**	**3.56 ± 0.32**	**0.76 ± 0.00**
150	**1.37**	**1.29 ± 0.06**	**47.17 ± 1.49**	**3.51 ± 0.26**	**0.78 ± 0.00**

Table S 3. Solar cell performance for thermal annealing optimization of **SAM-72:PC$_{71}$BM** devices.

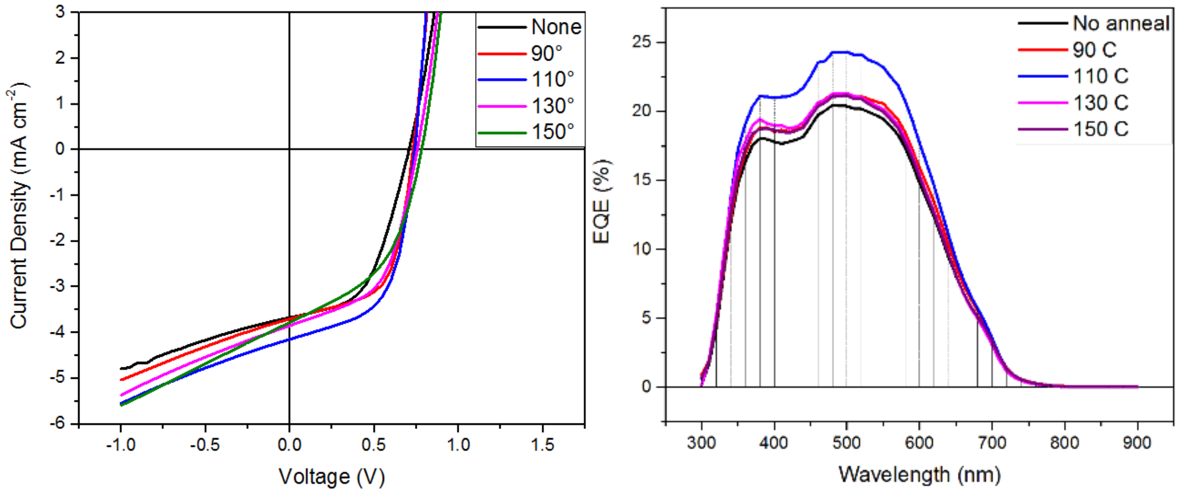

(a) (b)

Figure S 5. (a) Current-voltage plots and (b) EQE spectra for thermal annealing optimization of **SAM-72:PC$_{71}$BM** devices.

SVA time (s)	Best PCE (%)	PCE (%)	FF (%)	J_{sc} (mA/cm^2)	V_{oc} (V)
No SVA	**2.10**	**2.00 ± 0.08**	**55.66 ± 2.29**	**4.81 ± 0.28**	**0.75 ± 0.01**
10	**2.16**	**1.98 ± 0.18**	**65.02 ± 0.93**	**4.27 ± 0.40**	**0.72 ± 0.00**
30	**2.31**	**2.13 ± 0.15**	**64.24 ± 1.79**	**4.60 ± 0.31**	**0.72 ± 0.00**
60	**1.62**	**0.97 ± 0.58**	**46.43 ± 12.06**	**2.71 ± 1.18**	**0.73 ± 0.01**

Table S 4. Solar cell performance for solvent vapor annealing optimization of **SAM-72:PC$_{71}$BM** devices using CS_2.

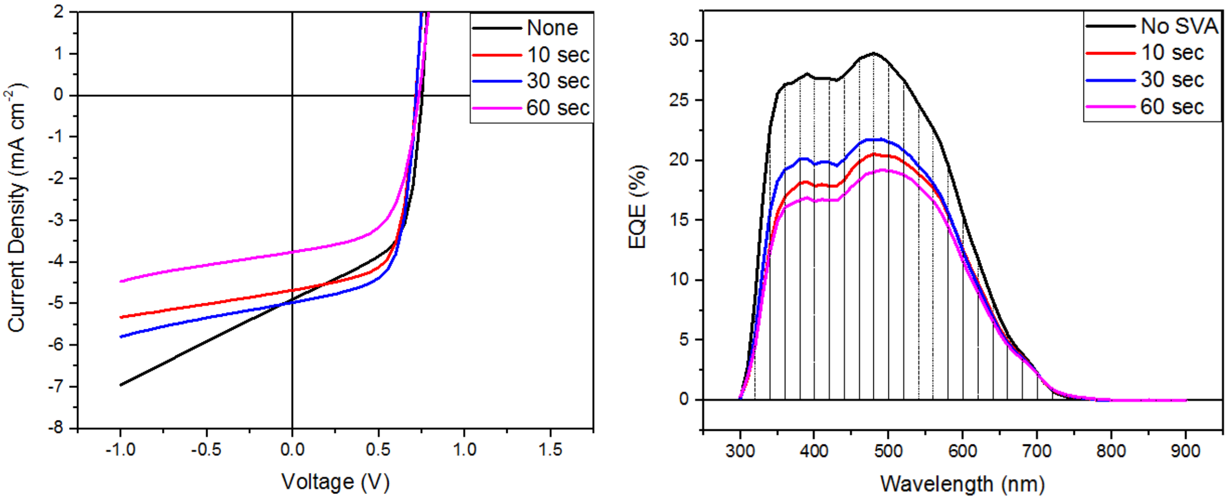

(a) (b)

Figure S 6. (a) Current-voltage plots and (b) EQE spectra for solvent vapor annealing optimization of **SAM-72:PC$_{71}$BM** devices using CS_2.

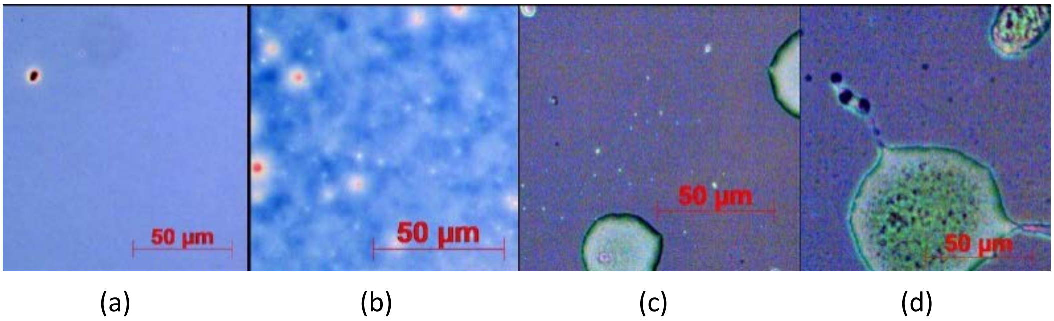

Figure S 7. Optical microscope images of **SAM-72:PC$_{71}$BM** films with 0v% (a), 1v% (b), 3v% (c), and 5v% (d) DIO added to precursor solution.

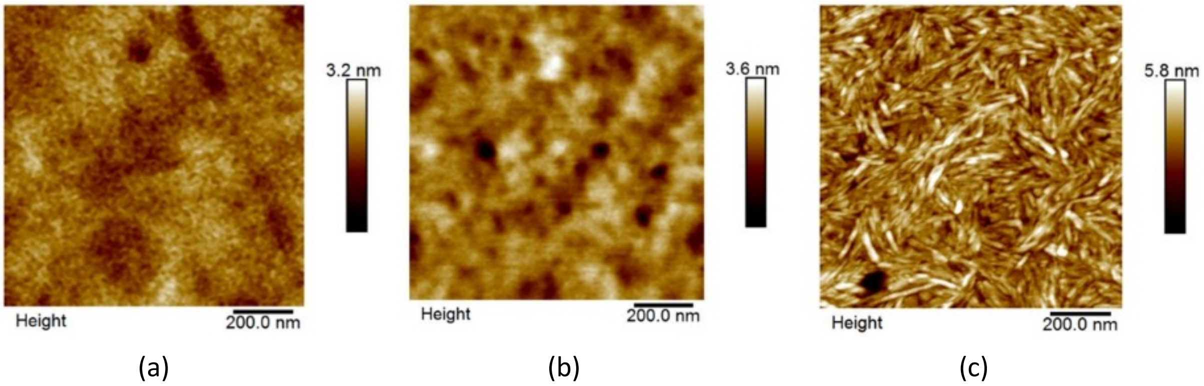

Figure S 8. Height AFM images of **SAM-72:PC$_{71}$BM** films under different processing methods: (a) as cast film with optimized donor and acceptor ratio, (b) thermal annealed film, and (c) film that was both thermally and solvent vapor annealed.

1H NMR and 13C NMR spectra

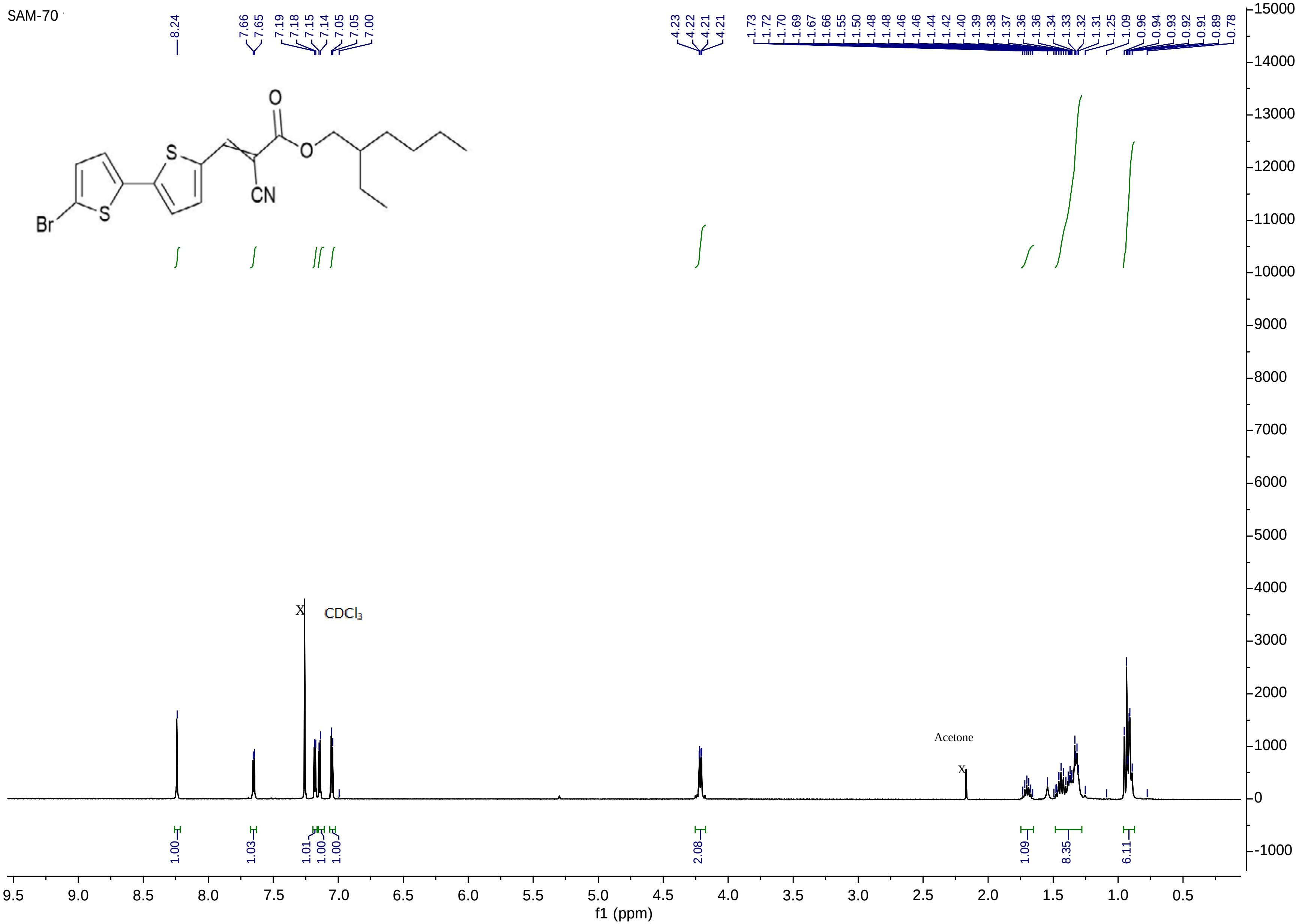

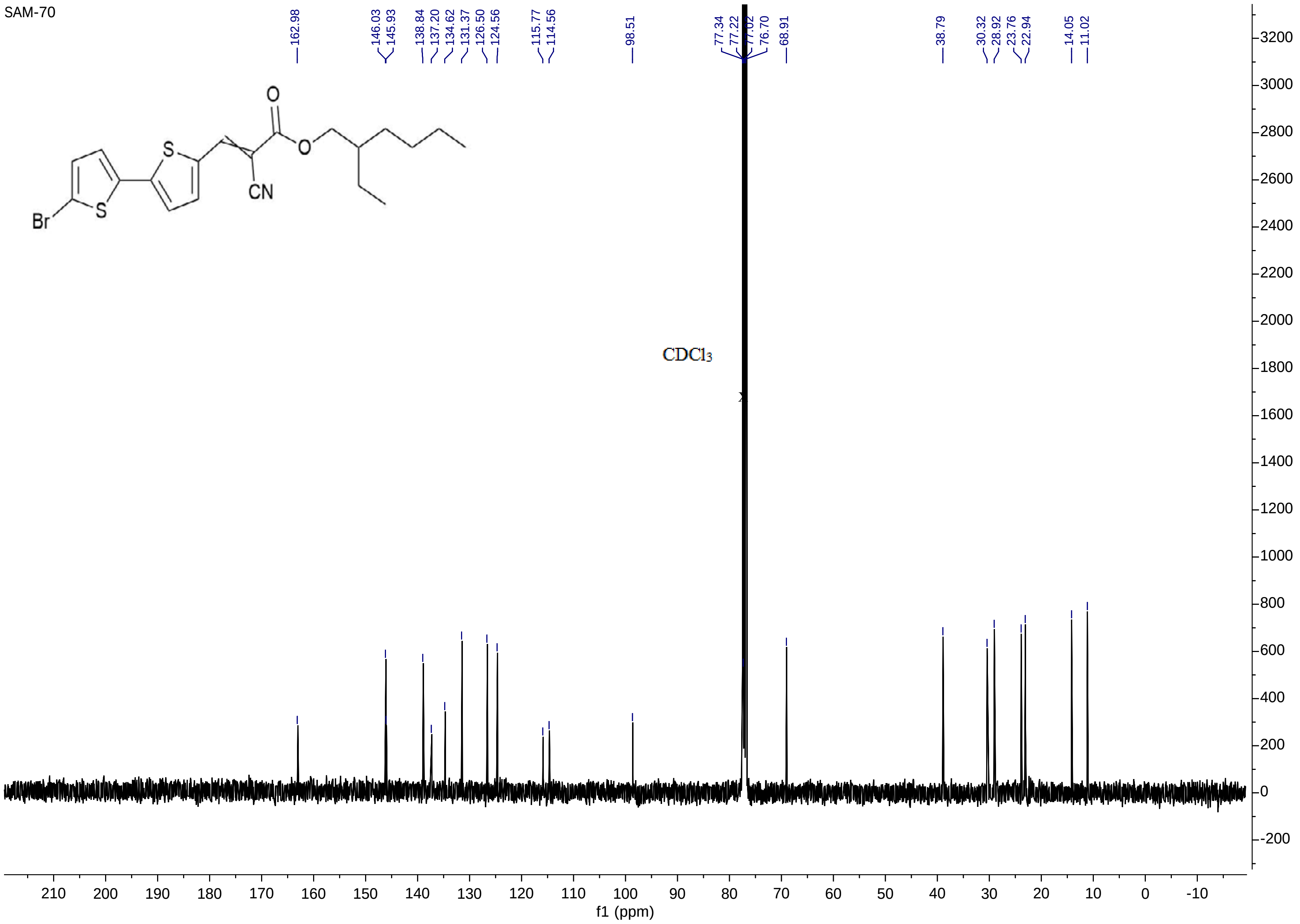
SAM-70
162.98
146.03
145.93
138.84
137.20
134.62
131.37
126.50
124.56
115.77
114.56
98.51
77.34
77.22
77.02
76.70
68.91
38.79
30.32
28.92
23.76
22.94
14.05
11.02
CDCl3
f1 (ppm)

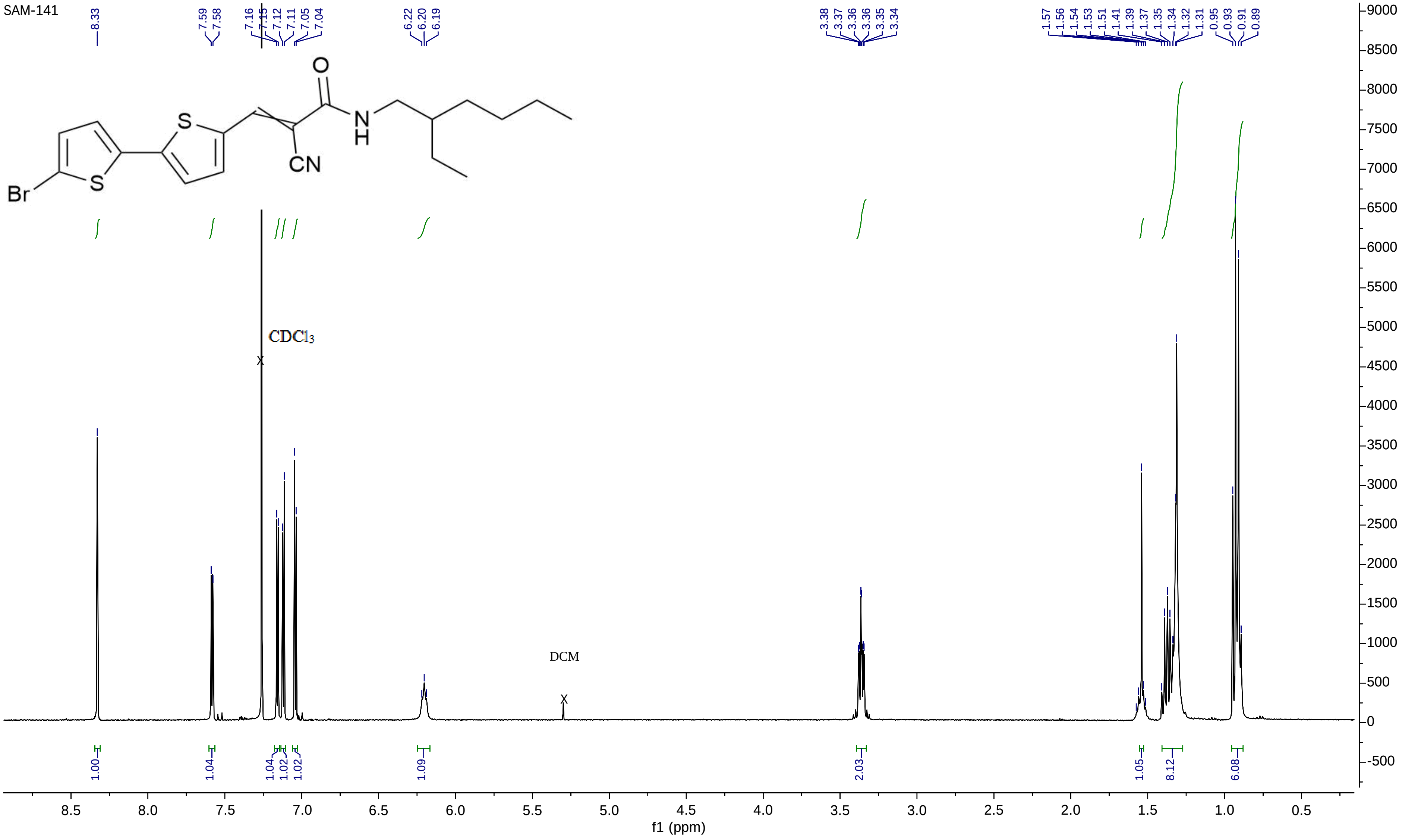
SAM-141
CDCl3
DCM
f1 (ppm)

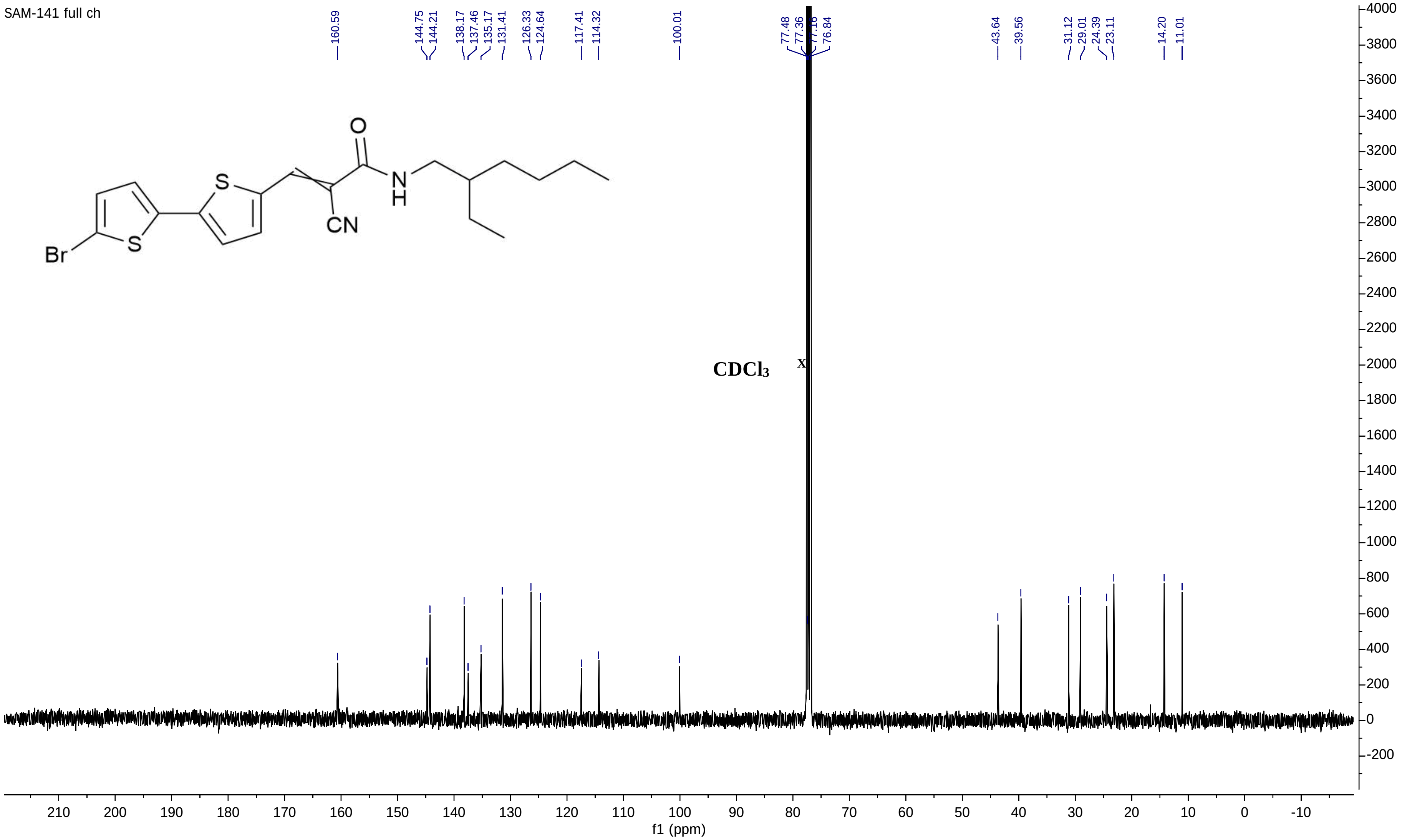
SAM-141 full ch
160.59
144.75
144.21
138.17
137.46
135.17
131.41
126.33
124.64
117.41
114.32
100.01
77.48
77.36
77.16
76.84
43.64
39.56
31.12
29.01
24.39
23.11
14.20
11.01
O
S
N
H
S
CN
Br
CDCl3
x
f1 (ppm)

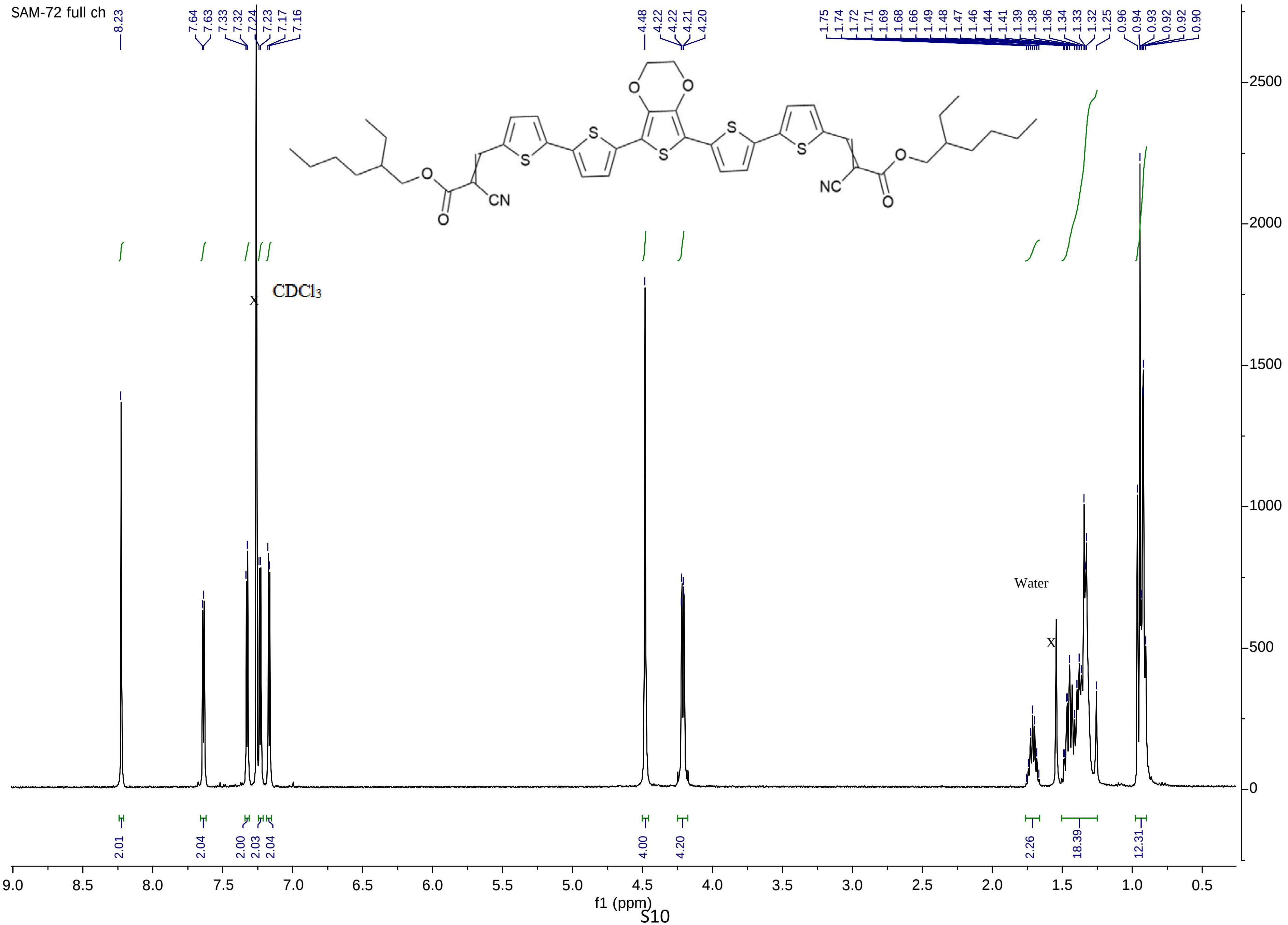
SAM-72 full ch
CDCl3
Water
NC
CN
f1 (ppm)

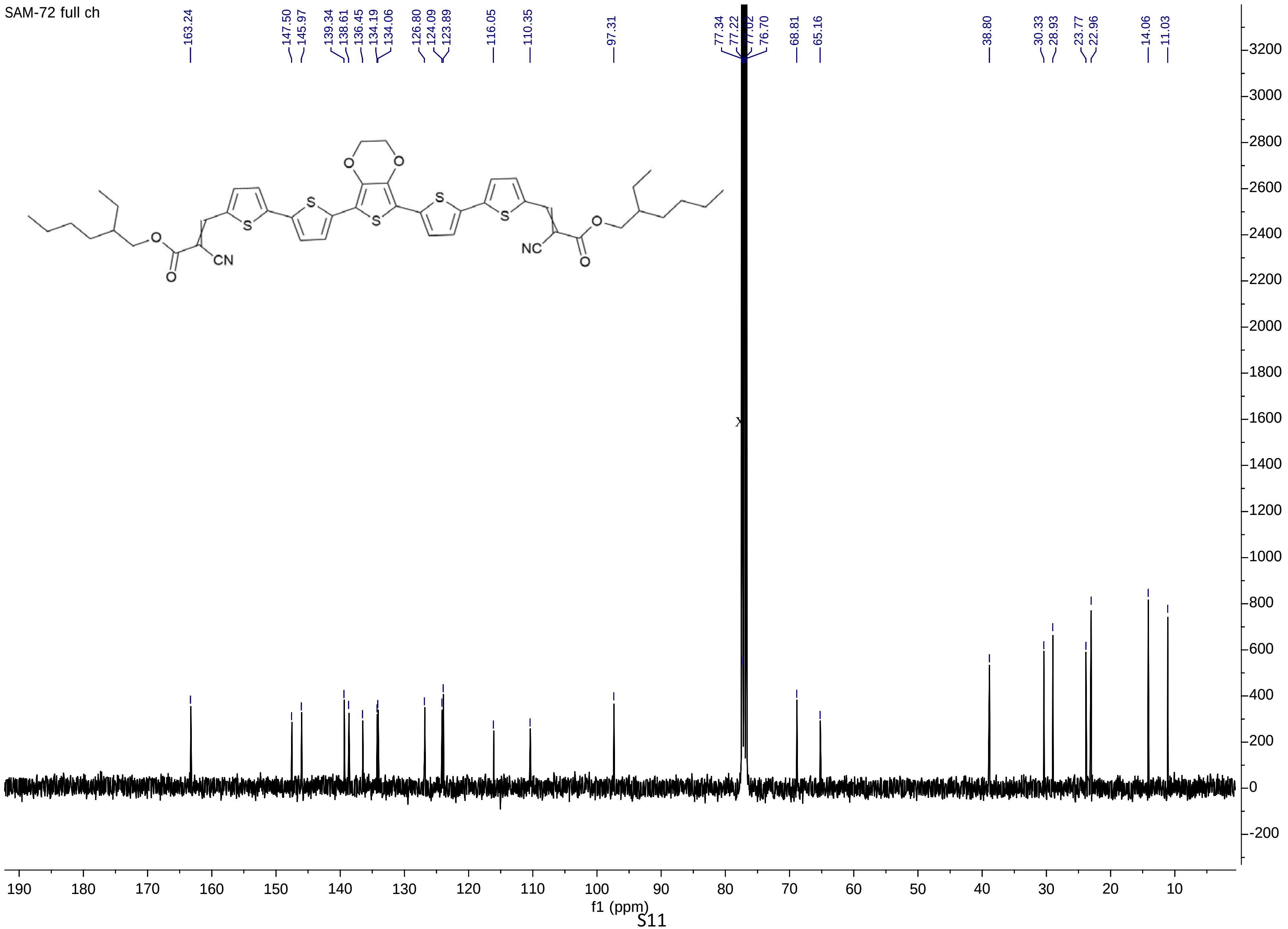
SAM-72 full ch
163.24
147.50
145.97
139.34
138.61
136.45
134.19
134.06
126.80
124.09
123.89
116.05
110.35
97.31
77.34
77.22
77.02
76.70
68.81
65.16
38.80
30.33
28.93
23.77
22.96
14.06
11.03
f1 (ppm)

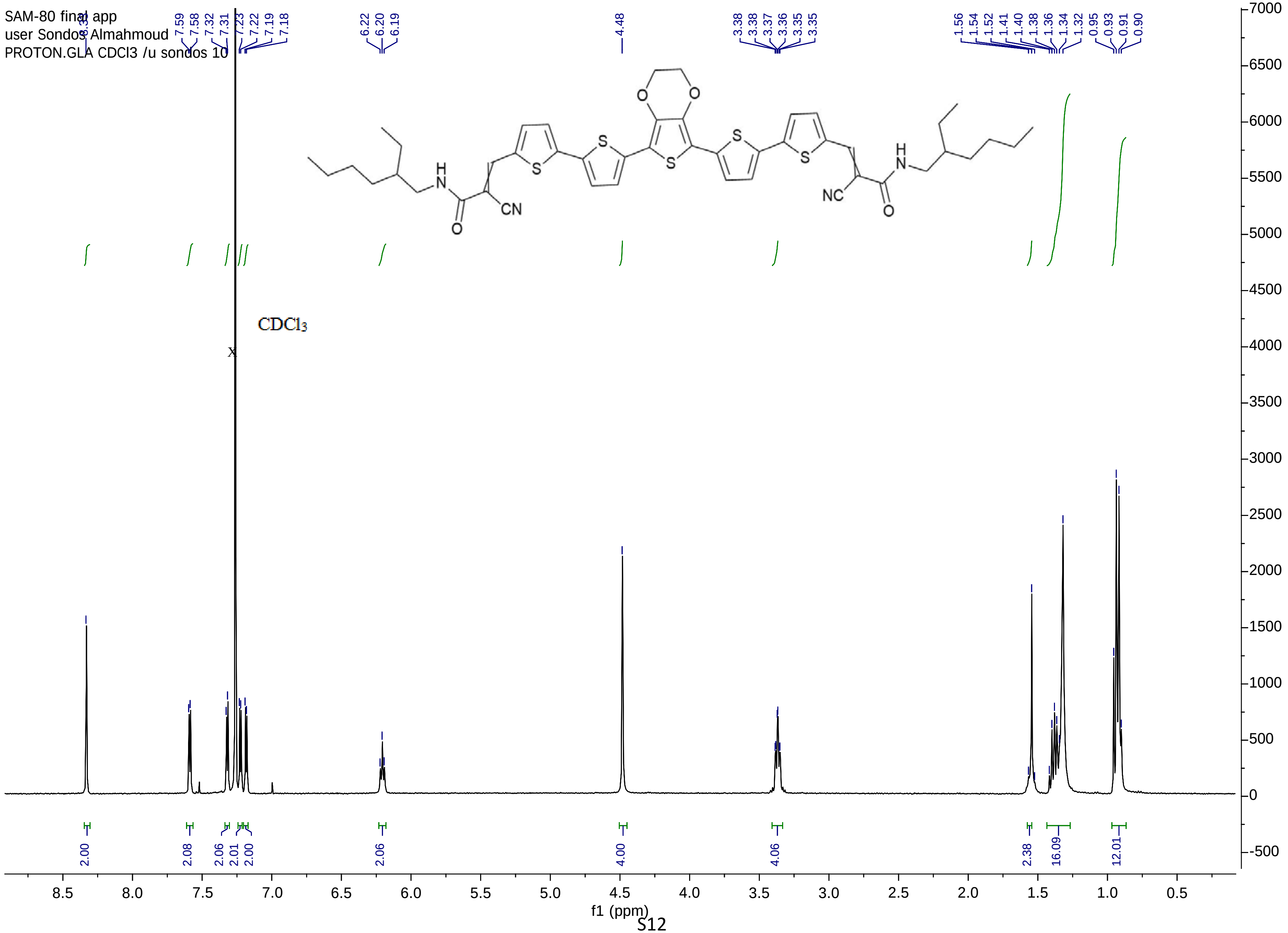
SAM-80 final app
user Sondos Almahmoud
PROTON.GLA CDCl3 /u sondos 10
7.59 7.58 7.32 7.31 7.23 7.22 7.19 7.18
6.22 6.20 6.19
4.48
3.38 3.38 3.37 3.36 3.35 3.35
1.56 1.54 1.52 1.41 1.40 1.38 1.36 1.34 1.32 0.95 0.93 0.91 0.90
CDCl3
2.00 2.08 2.06 2.01 2.00 2.06 4.00 4.06 2.38 16.09 12.01
f1 (ppm)

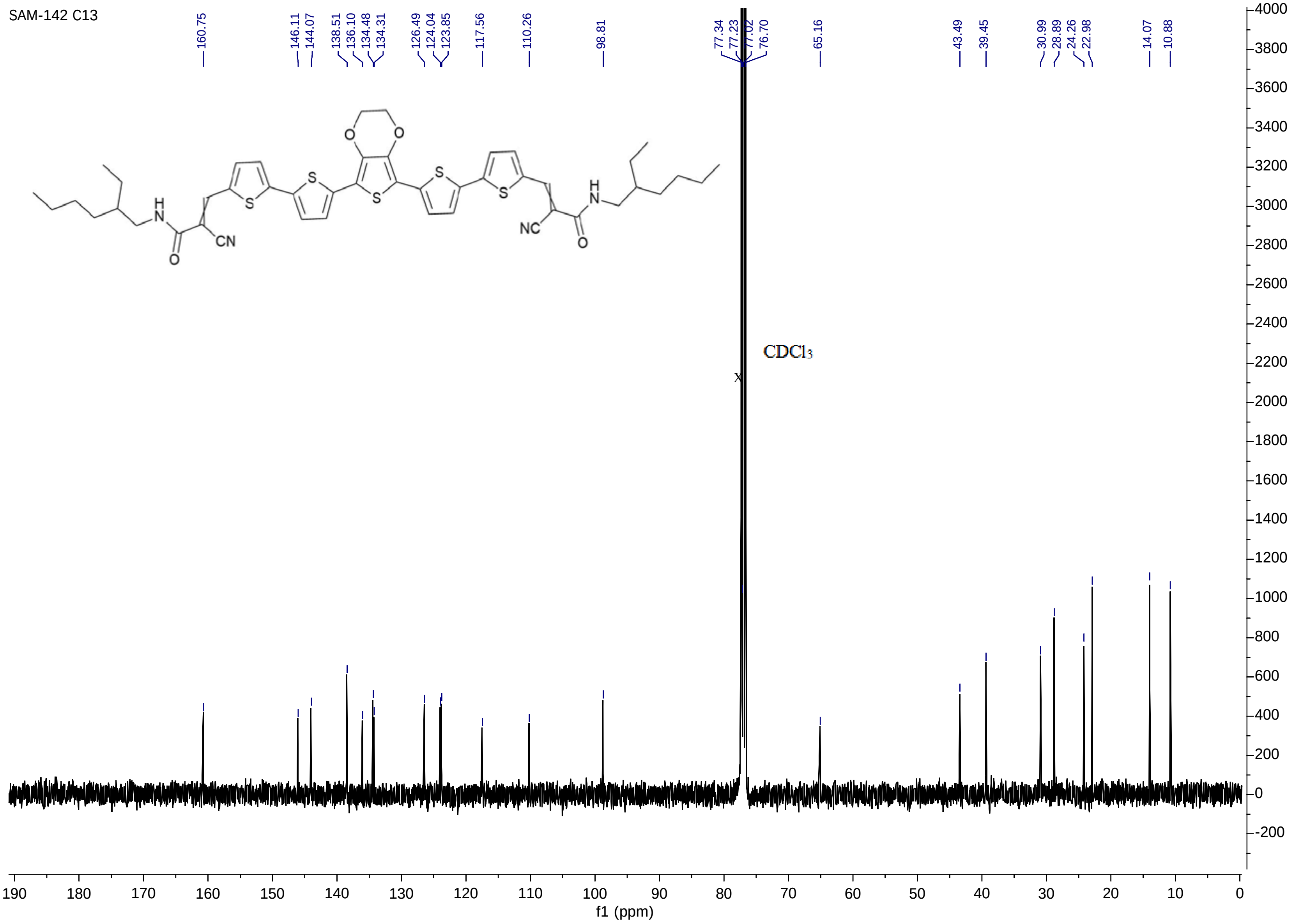
SAM-142 C13
160.75
146.11
144.07
138.51
136.10
134.48
134.31
126.49
124.04
123.85
117.56
110.26
98.81
77.34
77.23
77.02
76.70
65.16
43.49
39.45
30.99
28.89
24.26
22.98
14.07
10.88
CDCl3
x
f1 (ppm)
